# Tumor Reduction with Pazopanib in a Patient with Recurrent Lumbar Chordoma

**DOI:** 10.1155/2018/4290131

**Published:** 2018-04-10

**Authors:** Maurício Fernando Silva Almeida Ribeiro, Micelange Carvalho de Sousa, Samir Abdallah Hanna, Marcos Vinicius Calfat Maldaun, Ceci Obara Kurimori, Luiz Guilherme Cernaglia Aureliano de Lima, Romulo Loss Mattedi, Rodrigo Ramella Munhoz

**Affiliations:** ^1^Hospital Sírio Libanês, São Paulo, SP, Brazil; ^2^Oncology Service, Instituto do Câncer do Estado de São Paulo, Universidade de São Paulo, São Paulo, SP, Brazil

## Abstract

**Introduction:**

Chordomas are rare malignancies of bone origin that occur in the axial skeleton, typically the skull base and lumbar/sacral regions. Although often classified as low-grade neoplasms, its locally infiltrative behavior may result in significant morbidity and mortality. Optimal surgical resection may be curative, but up to 50% of the cases relapse within 5 years, and currently there are no systemic treatments approved in this setting. A large proportion of these tumors express stem-cell factor receptor (c-KIT) and platelet-derived growth factor receptors (PDGFRs), providing a rationale for the use of tyrosine-kinase inhibitors (TKIs).

**Case report:**

A 27-year-old male presented with recurrent chordoma of the lumbar spine 4 years after initial diagnosis. Salvage therapies in the interval included repeat resections and radiation therapy. He ultimately developed multifocal recurrence not amenable to complete excision or reirradiation. A comprehensive genomic profiling assay was performed and revealed nondrugable alterations. Decision was made to proceed with systemic treatment with pazopanib 800 mg/day, resulting in tumor reduction (−23.1% reduction in size) and prolonged disease control.

**Conclusion:**

For this patient with a multiple recurrent chordoma and limited treatment options, pazopanib resulted in sustained clinical benefit following initial tumor reduction.

## 1. Introduction

Chordomas are uncommon neoplasms that originate from embryonic remnants of notochord and account for only 1–4% of all bone tumors. They predominate in men around the sixth decade of life, and have an approximate incidence of 0.1–0.8 per 1,000,000 individuals per year [1, 2]. Approximately 50% of the cases arise from the sacrum, 30% from skull base, and 20% from the vertebral column, most of which are diagnosed at advanced stages due to the presence of nonspecific symptoms (or their total absence) in early tumors. Three histological subtypes are described: conventional, chondroid, and dedifferentiated, the latter being the rarest and most aggressive subtype [3, 4]. Most chordomas present high levels of expression of brachyury, a key transcription factor for the development of notochord, being silenced after embryonic development. Reactivation of the brachyury gene, associated with its hyperexpression, is considered a driver alteration in the development and progression of these tumors. In addition, the presence of positive immunostaining for brachyury has aided in the differential diagnosis from other neoplasms with primary site and histologic similarities [5].

*En bloc* resection with negative margins remains the treatment of choice [1, 6]. However, in up to 50% of the cases, the magnitude of its extension makes radical surgery difficult. The use of radiation therapy for patients with chordomas is still a matter of debate, especially considering the need for high doses of radiation (60–80 Gy) in the tumor and the potential toxicities related to the treatment. Nevertheless, adjuvant radiation therapy is often used for patients undergoing suboptimal surgical procedures or for those with recurrent disease; advanced radiation techniques, including stereotactic radiosurgery and proton therapy, are being explored. Local recurrence is the main adverse prognostic factor in chordomas, making regional control challenging, given the limited possibilities of salvage therapies. Increased toxicity in previously irradiated structures (i.e., rectum and spinal cord) may be a limiting factor for reirradiation, and even repeat surgical procedures of already irradiated regions may be problematic [1, 4, 5].

For patients with advanced disease or refractory to conventional therapeutic modalities, there are no systemic treatments approved for clinical use to date. It is estimated that approximately 75–90% of chordomas exhibit positive immunostaining for tyrosine kinase receptors involved in signaling pathways related to cell proliferation and angiogenesis, such as the stem-cell factor receptor (C-KIT), platelet-derived growth factor receptor *α* (PDGFR-*α*), platelet-derived growth factor receptor *β* (PDGFR-*β*), and vascular endothelial growth factor (VEGF) [7, 8]. Based on this rationale, several authors have interrogated the activity of tyrosine kinase inhibitors (TKIs) for the blockade of these signaling pathways (i.e., imatinib, sorafenib, and sunitinib) in previously treated patients, with variable rates of disease control [7, 9–14]. However, no randomized trials have been conducted, and conclusions are hampered by the lack of control arms and bias inherent to a disease that may exhibit an indolent course. Despite the widespread use in other sarcoma subtypes, the activity of pazopanib in chordomas is poorly characterized. Lipplaa et al. described disease control in 2/4 patients treated with pazopanib 800 mg daily, with PFS intervals of 14 and 15 months [15].

In this report, we describe the case of a 27-year-old male diagnosed with a lumbar spine conventional chordoma with multiple recurrences following surgery and radiotherapy, who presented an important clinical and radiological response to pazopanib.

## 2. Case Report

A 27-year-old white male presented in December 2015 for evaluation of a relapsed conventional chordoma arising from the lumber spine. His diagnosis dated back to August 2013, when he underwent additional evaluation due to a recurrent lower back pain. Magnetic resonance imaging (MRI) of the lumbar spine and sacrum revealed a solid expansive lesion on L2 topography, with an extensive epidural soft tissue component, infiltrating the bone marrow and determining compression of the dural sac. At that time, a core biopsy was performed, which revealed a lesion consistent with a grade 2 chordoma with positive immunostaining for pan-cytokeratin (AE1/AE3), S-100 protein, epithelial membrane antigen (EMA), and Ki-67 index of 20%. The immunohistochemical assessment of brachyury was not conclusive. Three months after the diagnosis, the patient underwent L2 vertebrectomy with negative microscopic margins that confirmed the diagnosis of chordoma of the lumbar spine.

**Figure 1:**
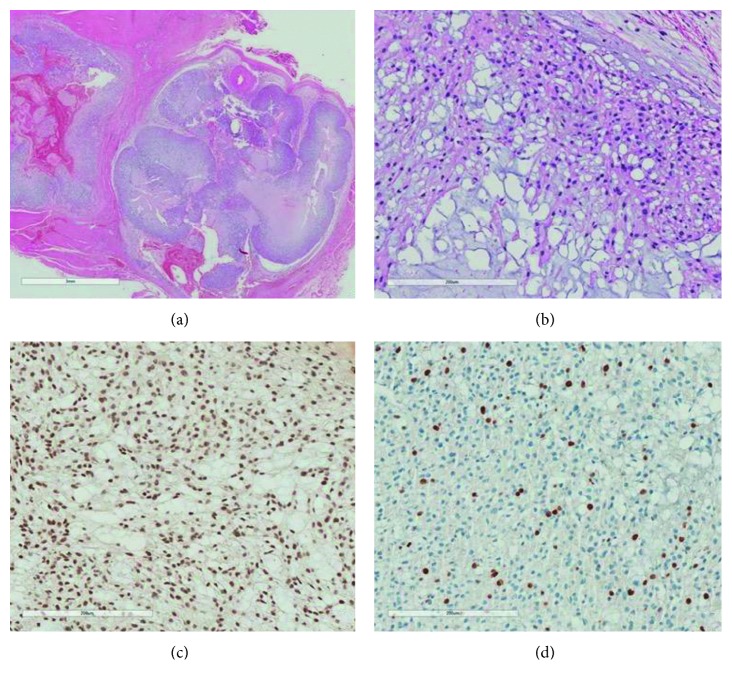
Histopathologic and immunohistochemical evaluation. (a) Chordoma with expansive invasion (hematoxylin-eosin–HE, 10x). (b) Epithelioid eosinophilic cells in myxoid matrix (HE, 200x); (c) INI-1 positive/preserved expression in neoplastic cells (immunohistochemistry, 200x); (d) Ki-67 positive in neoplastic cells (immunohistochemistry, 200x).

Surgical treatment was followed by adjuvant intensity-modulated radiotherapy (IMRT) to the tumor bed (45 Gy in 25 fractions), and he was then managed expectantly until September/2015, when he presented with disease relapse. MRI of the lumbosacral spine showed multiple lesions of cystic appearance and of small dimensions distributed in paravertebral soft parts at the level of L2, in the right psoas muscle and in the vertebral bodies of T11 and T12, measuring up to 2.2 cm, the largest diameter, without significant contrast enhancement. A new biopsy was then performed and reviewed by a reference pathologist, with histological confirmation of atypical chordoma. The neoplasia showed an expansive and multilobulated pattern of invasion and aspect of chordoma, with epithelioid and eosinophilic cells in the chondromyxoid matrix background. The mitosis figures were observed up to 04/10 high-power fields (HPFs) (Figures 1(a) and 1(b)). There were no areas of dedifferentiation (INI-1 intact). Despite the morphological and immunoexpression of cytokeratins, EMA and vimentin in neoplastic cells, expression of brachyury in the neoplasia was not detected (Figures 1(c) and 1(d)). Of note, even though it is an unusual finding, negative brachyury can be observed in rare cases, predominantly solid chordomas comprising more atypical cells with mitoses and necrosis [16].

A repeat MRI performed after 1 month revealed an increase in previously existing lesions, compression of the dural sac and medullary cone by a lesion extending from T11 to T12, and additional noncontiguous lesions at the level of L2 and multiple nodules measuring up to 1 cm in the right posterior abdominal musculature. In view of the spinal cord compression, a new surgical approach was recommended, and the patient underwent a T11-T12 level partial resection/decompression.

**Figure 2:**
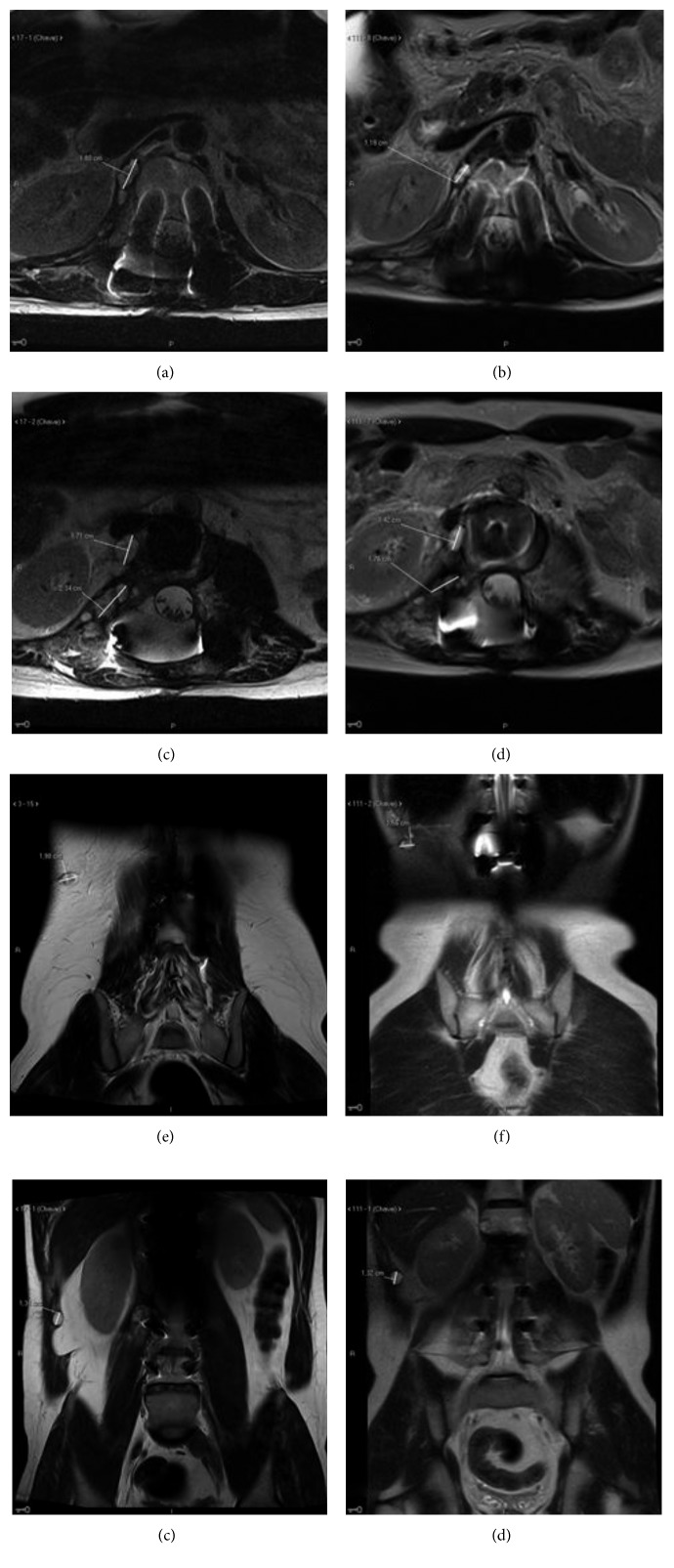
Baseline and posttreatment imaging showing multifocal recurrence of spinal chordoma and tumor reduction following treatment with pazopanib. In the left column (a, c, e, and g): baseline lumbar spine MRI. In the right column (b, d, f, and h): follow-up abdominal MRI.

Eight months later, disease progression was once again documented in the thoracolumbar transition and the patient underwent another surgical procedure, with partial resection of the epidural lesion between T11 and T12, as well as the additional lesions between L1-L2 and L3. One month later, the control MRI showed an increase in the dimensions of the preexisting lesions in the lumbar spine and in the abdominal musculature, without new foci. The patient was then treated with image-guided radiotherapy (IGRT) at the dose of 54 Gy in the surgical bed and in regions of gross disease (bilateral paravertebral between T10 and S1). One month after the end of the radiotherapy treatment, a new MRI was performed (Figures 2(a)–2(g)), which indicated disease progression outside the radiated area.

Considering the lack of salvage therapies available in this scenario, a FoundationOne®Heme genomic profiling assay was requested, which revealed 5 nondrugable genomic alterations: *ATM* Q2593^∗^ mutation, *ARID1A* S2149fs^∗^47 mutation, *CDKN2A/B* loss, *EP300* L415P mutation (subclonal), and *MLL3* Y306^∗^ mutation. *INI-1/SMARCB1* was included in the panel and showed no mutations. Despite the rationale for the use of poly (ADP-ribose) polymerase (PARP) inhibitors in patients with loss-of-function *ATM* mutations, there were no ongoing clinical trials for those alterations at that time.

Given the limited therapeutic options in the scenario, systemic treatment with pazopanib 800 mg/day was recommended. The patient developed grade 3 neutropenia, requiring dose reduction (400 mg daily, followed by 400 mg alternated with 800 mg per day), with subsequent good tolerance.

Restaging scans were performed following approximately 3 months of treatment with pazopanib, showing a reduction in size of previously described lesions in anterior paravertebral region, right psoas, and abdominal musculature, and stable dimensions of remaining foci of disease; no new lesions were characterized (Figures 2(b)–2(g)). The sum of the target lesions measurements showed a 23.1% reduction between baseline and posttreatment images (Table 1). Although reduction in target lesions was observed, accompanied by clinical improvement, we could not confirm a partial response according to the Response Evaluation Criteria in Solid Tumors 1.1 (RECIST 1.1). Focal growth in the form a paravertebral lesion was noted after 9 months of therapy, accompanied by sustained control of additional target lesions. Decision was made to proceed with salvage surgical resection of the area of progression. Due to the stability of nonexcised foci of disease, treatment with pazopanib was resumed after the surgery, and the patient continues to endure prolonged clinical benefit more than 15 months after treatment initiation.

## 3. Discussion

We report a case of a patient with a multiple recurrent lumbar spine chordoma initially treated with gross total resection with wide margins followed by adjuvant radiation therapy. Despite multimodal therapy, consisting of multiple surgical resections and reirradiation, the patient experienced locoregional disease progression and distant metastasis. Prolonged disease control following initial tumor reduction was achieved with the introduction of pazopanib, suggesting a role of this agent in the management of this challenging disease.

Although considered low-grade neoplasms that usually exhibit an indolent clinical course, conventional chordomas have a significant negative impact on patients' quality of life and may result in pronounced morbidity [17]. Complete *en bloc* surgical resection with wide margins remains the standard treatment; nevertheless, recurrences may develop in up to 50% of the cases. Radiotherapy is often used in patients presenting with locally advanced disease not amenable to complete excision or in scenarios in which surgery with negative margins is not feasible. For patients who fail standard approaches, treatment alternatives are limited to “salvage” re-resection with curative intent in a very small percentage of patients who present with isolated lesions, long disease-free interval, good performance status, and acceptable morbidity; for those with multifocal recurrences, a history of piecemeal resection, tumor rupture, or prior treatment with high-dose RT, curative-intent therapies are definitely not recommended based on most recent guidelines from the Chordoma Global Consensus Group [18]. The lack of systemic agents capable of altering the course of the recurrent disease has made the management of these patients even more difficult [1, 3, 4].

Based on the frequent expression of markers associated with aberrant activation of signaling pathways related to cell proliferation and angiogenesis, including C-KIT, PDGFR-*α*/*β* and VEGF [7, 8], multiple attempts to use targeted therapies for this orphan disease are described in the literature. Casali et al. reported clinical benefit in 4/5 cases of imatinib-treated chordoma of the sacrum [9]. A prospective, multicenter, phase II, single-arm study evaluated the efficacy of imatinib 400 mg bid in 56 cases of chordomas with PDGFR-*β* overexpression, with clinical benefit demonstrated in 64% of patients and median PFS of 9 months [10]. In another phase II trial, which included several neoplasms (with positive immunostaining for imatinib-blocking receptors, despite the absence of activating mutations or rearrangements associated with PDGFR-*β*), stable disease was observed in 80% of the cases (4/5 cases) of the chordoma cohort [11]. The largest retrospective study evaluated 80 cases of recurrent chordomas treated with molecular target therapies (imatinib in 77% of the cases), showing median PFS and OS of 9.4 months and 4.4 years, respectively. Disease control rate was 78.8%, with only 6.3% of the patients achieving an objective response. Despite the low response rates observed, nearly 42% of the patients experienced symptomatic improvement, which was associated with longer PFS and duration of treatment [19].

In imatinib-pretreated patients, a study performed by Stacchiotti et al. comprising 10 cases of chordoma (9 arising in the sacrum and 1 in the skull-base) reported a PFS rate at 6 months of 87% with the combination of imatinib and sirolimus, supporting the role of the mammalian target of rapamycin (mTOR) pathway as a mechanism of resistance to imatinib in these tumors [7, 12]. The use of sunitinib 37.5 mg daily was also evaluated in a multicenter phase II trial that included non-GIST sarcomas. Among 9 cases of advanced chordoma, PFS rates of 20% and 12% at 16 and 24 weeks were observed, respectively, despite no objective responses [13]. A distinct TKI, sorafenib, was evaluated in a multicenter phase II study, coordinated by the French Sarcoma Group (GSF/GETO). Twenty-seven patients with chordoma were treated with sorafenib 800 mg daily, with 73% of patients being progression-free at 9 months and 86.5% alive at 12 months [14]. Using agents aiming at epidermal growth factor receptor (EGFR) blockade, Asklund et al. reported durable disease stability in 3 patients with recurrent chordomas treated with the combination of erlotinib and bevacizumab, with disease control intervals ranging from 24 to 54 months [2]. Based on the same rationale, the efficacy of lapatinib 1500 mg/day was interrogated in a phase II study in patients with chordoma with hyperexpression of EGFR and HER-2, which resulted in stable disease in 15 of the 18 patients, with median PFS and median OS of 8 and 25 months, respectively [20].

More recently, the development of a recombinant *Saccharomyces cerevisiae*-based vaccine encoding brachyury (GI-6301) brought a new insight into the treatment of chordomas [21]. In the phase I dose-escalation study, 34 patients with several solid tumors refractory to standard therapies, including 11 chordomas, were treated with GI-6301; objective response was observed in one patient within the chordoma cohort, with one being considered not available for RECIST 1.1 criteria, and the remaining 9 patients being progression-free in 5 months. A double-blind, phase II study testing the association of GI-6301 with radiotherapy in locally advanced and unresectable chordomas is currently ongoing (NCT02383498). There is also an ongoing phase I study aimed at evaluating the combination of nivolumab with stereotactic radiosurgery versus exclusive stereotactic radiosurgery in locally advanced, relapsed, or metastatic chordomas (NCT02989636). Still regarding the combined blockade of the PDGFR-*β* and mTOR pathways in advanced chordomas, we wait for the outcomes of the phase II study testing the efficacy of imatinib and everolimus combination therapy (EudraCT 2010-021755-34).

In conclusion, chordomas are challenging neoplasms with an unpredictable clinical course and a high chance of relapse. Patients presenting with multiple recurrent disease bring an unmet need for systemic therapies. Despite the lack of randomized trials, this case report adds to the available literature suggesting a role for the use of pazopanib.

## Figures and Tables

**Table 1:** Size of target lesions (measurements of the largest diameter).

	Baseline	Follow-up
	Target lesions	
T1 paravertebral	2.34 cm (Figure 2(c))	1.76 cm (Figure 2(d))
T2 abdominal wall	1.98 cm (Figure 2(e))	1.56 cm (Figure 2(f))
Sum	4.32 cm	3.32 cm (−23.1%)
	Nontarget lesions	
T3 paravertebral	1.80 cm (Figure 2(a))	1.18 cm (Figure 2(b))
T4 paravertebral	1.71 cm (Figure 2(c))	1.42 cm (Figure 2(d))
T5 abdominal wall	1.34 cm (Figure 2(g))	1.32 cm (Figure 2(h))
